# *Neospora caninum* Infection in Marine Mammals Stranding in Northeastern Pacific Ocean Region

**DOI:** 10.3201/eid3202.251507

**Published:** 2026-02

**Authors:** Stephen A. Raverty, Pádraig Duignan, Dyanna M. Lambourn, Paul Cottrell, Verena A. Gill, Pamela Tuomi, Lorraine Barbosa, Brendan Cottrell, Spencer L. Magargal, Amanda K. Gibson, Elizabeth R. Zhang, Michael E. Grigg

**Affiliations:** Animal Health Center, British Columbia Ministry of Agriculture, Abbotsford, British Columbia, Canada (S.A. Raverty); The Marine Mammal Center, Sausalito, California, USA (P. Duignan, L. Barbosa); University of Calgary, Veterinary Medicine, Calgary, Alberta, Canada (P. Duignan); Washington Department of Fish and Wildlife, Lakewood, Washington, USA (D.M. Lambourn); Department of Fisheries and Oceans, Vancouver, British Columbia, Canada (P. Cottrell); US Fish and Wildlife Service, Anchorage, Alaska, USA (V.A. Gill); Alaska SeaLife Center, Seward, Alaska, USA (P. Tuomi); McGill University, Montreal, Quebec, Canada (B. Cottrell); National Institutes of Health, Bethesda, Maryland, USA (S.L. Magargal, A.K. Gibson, E.R. Zhang, M.E. Grigg)

**Keywords:** *Neospora caninum*, parasites, marine mammals, Pacific Ocean, protozoan pathogen, neosporosis, sea otter, sea lion, seal

## Abstract

We used immunohistochemistry and PCR to identify *Neospora caninum* in 6 infected marine mammal species, including 2 pups, that stranded in the northeastern Pacific Ocean. Our findings suggest the expansion of this parasite’s host range to marine mammals, underscoring the effect of terrestrial pathogens that flow from land to sea.

Researchers using immunohistochemistry and PCR-DNA sequencing analyses have confirmed high infection rates of the protozoan parasites *Toxoplasma gondii* and *Sarcocystis neurona*, often as mixed infections, in a range of stranded pinniped, cetacean, and mustelid species (*1*,*2*). Parasite transmission is closely linked to land-to-sea pathogen flow (*3*,*4*). We identified *Neospora caninum*, a protozoan pathogen known to affect reproductive fitness in livestock, in 6 different species of marine mammals stranded in the northeastern Pacific Ocean region. The emergence of *N. caninum* illustrates a third terrestrially sourced parasite (also referred to as a pollutagen) flowing from land to sea to infect marine mammals in this region. This parasite is distinct from previously reported *N. caninum*–like parasites that circulate between pinnipeds in a marine cycle (*5*).

In cattle, *N. caninum* is considered among the most efficient pathogens to be transmitted transplacentally (*6*). This pathogen is a major contributor of reproductive loss in the dairy industry worldwide. Dogs and wild canids, including foxes, wolves, and coyotes, are among the recognized hosts, both definitive and intermediate, for this parasite. Unlike most coccidian parasites that have a limited host range, *N. caninum* is increasingly detected in a wide array of terrestrial and avian species (*7*). Although reports have identified antibodies to *N. caninum* in prior serosurveys of marine mammals in Australia, Japan, and the United States (*8*–*10*), those assays were not validated for wildlife. Cross-reactivity with related coccidian parasites that commonly circulate between marine mammals in a marine cycle may have confounded interpretation of the results (*11*). We report 8 confirmed cases of *N. caninum* infection in 6 marine mammal species, including 2 pups and a pregnant female.

## The Study

**Figure 1:**
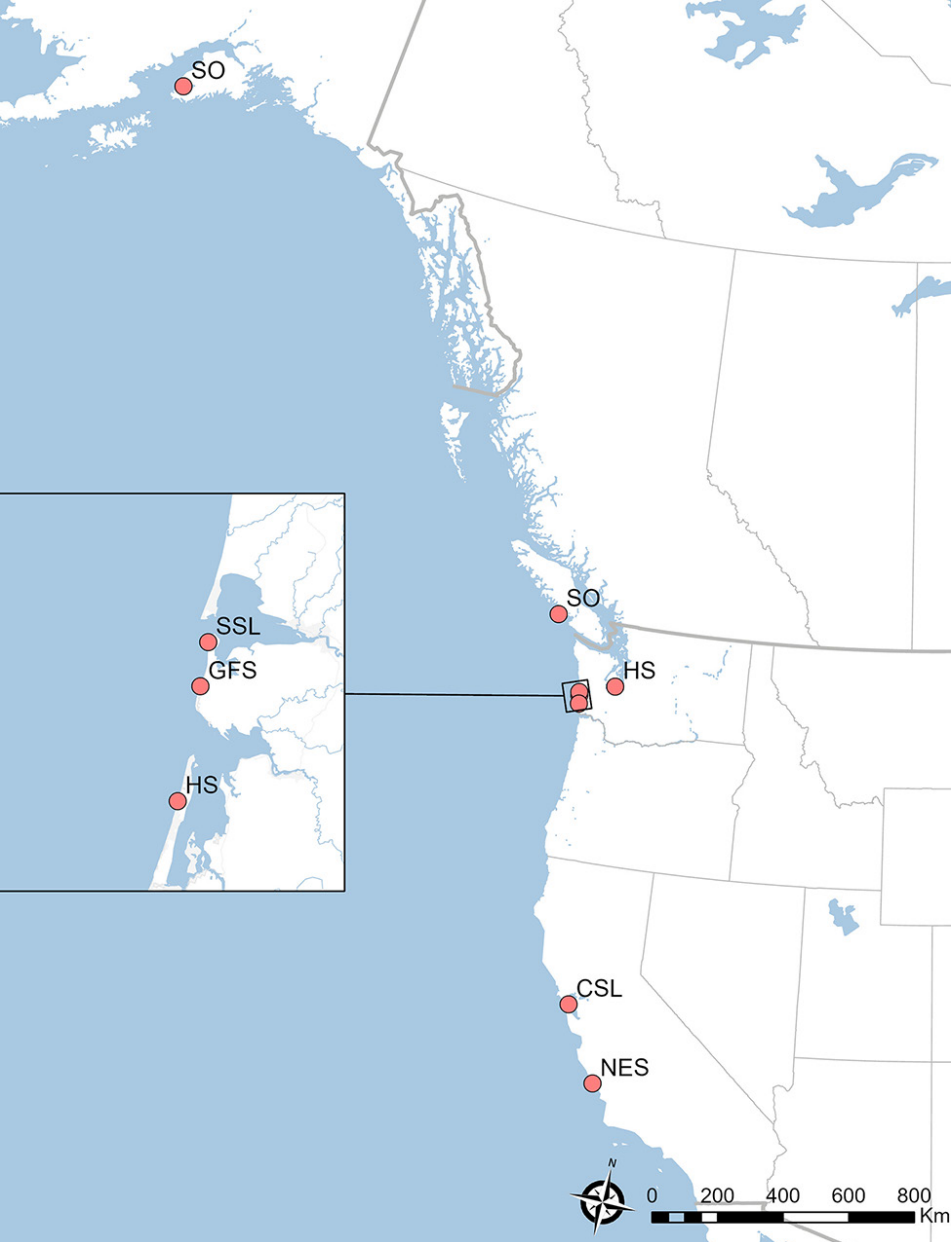
Stranding locations and animal species in study of *Neospora caninum* infection in marine mammals stranding in the northeastern Pacific Ocean region. NES, northern elephant seal (*Mirounga angustirostris*); CSL, California sea lion (*Zalophus californianus*); HS, harbor seal (*Phoca vitulina*); GFS, Guadalupe fur seal (*Arctocephalus townsendi*); SSL, Steller sea lion (*Eumetopias jubatus*); SO, sea otter (*Enhydra lutris*).

Throughout the northeastern Pacific region, local and regional marine mammal stranding networks respond to live stranded and dead marine mammals. In this case series, wildlife officials delivered a California sea lion (*Zalophus californianus*) for rehabilitation. Despite supportive care, the animal declined and was euthanized. We identified a solitary *N. caninum* infection using PCR, observing no discernible parasites by histopathology. As a result of that finding, we conducted a retrospective database analysis on 410 stranded marine mammals previously screened by PCR-DNA sequencing at the internal transcribed spacer (ITS) 1 marker. We identified 7 additional cases of *N. caninum* infection in another 5 species of marine mammals. The geographic range of the *N. caninum*–infected animals (Figure 1) suggested multiple points, rather than a single point source, of parasite exposure.

We documented the stranding location, recorded signalment, compiled morphometrics, and performed a necropsy for all 8 cases (Appendix Table). We also harvested representative tissues for histopathology and immunohistochemistry and froze samples for ancillary diagnostic studies. We conducted PCR-DNA sequencing using pan-Apicomplexan primers that amplify across the ITS1 region for speciation. We carried out immunohistochemistry for *N. caninum*, *T. gondii,* and *S. neurona* on available tissues from 4 PCR-positive animals, following previously reported protocols (*1*,*12*).

**Figure 2:**
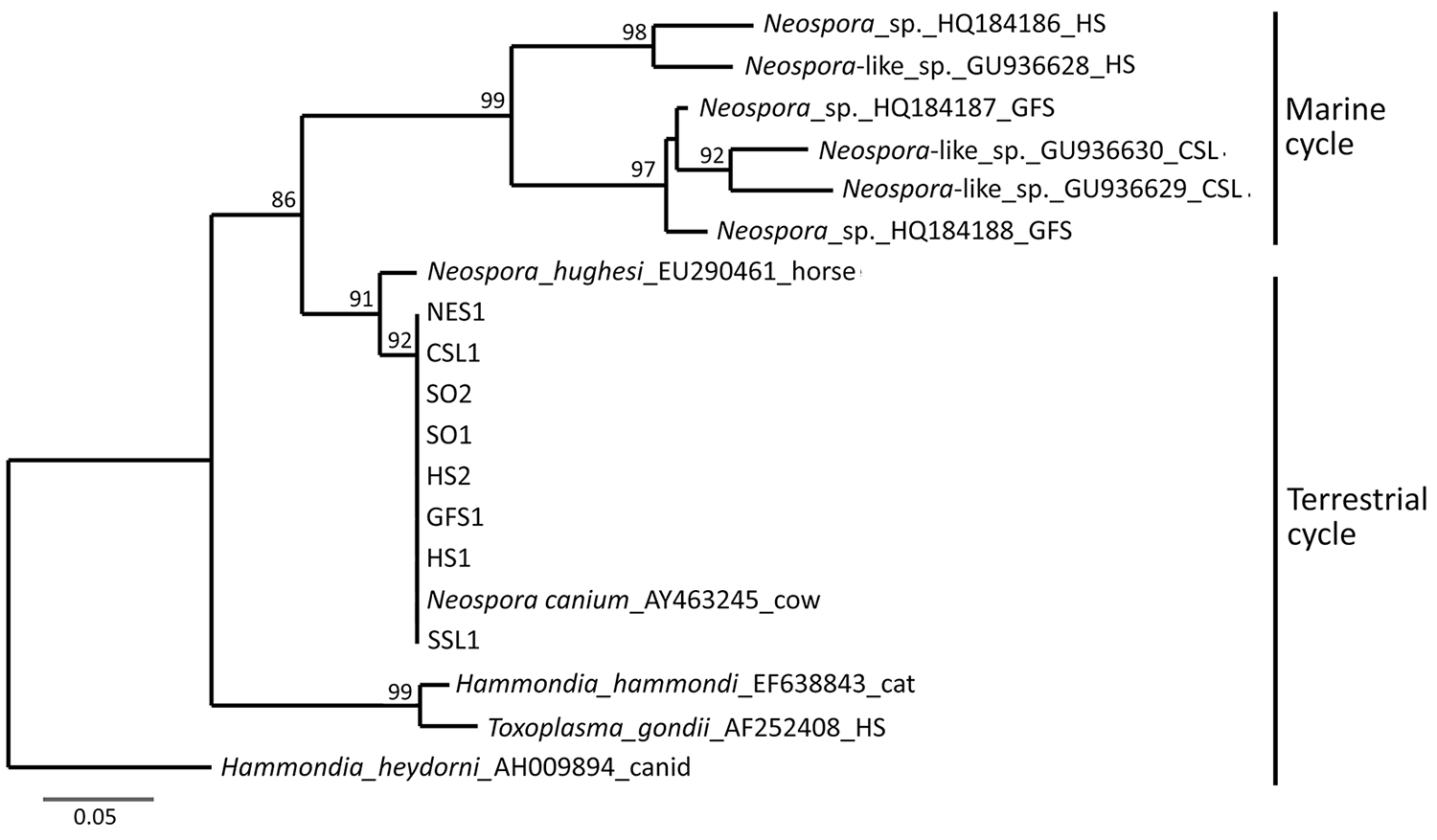
Phylogenetic relationships among *Neospora caninum*–like species that circulate in a marine cycle compared with terrestrial-sourced species from study of *N. caninum* infection in marine mammals stranding in the northeastern Pacific Ocean region. Neighbor-joining consensus tree shows marine strains that commonly infect pinnipeds and terrestrial strains (GenBank accession numbers PX529932–8) that transferred from land to sea to infect 5 species of pinnipeds. Evolutionary distances computed using the Tamura-Nei genetic distance model at the complete internal transcribed spacer 1 locus. Tree inferred using the outgroup sequence from *Hammondia heydorni* to root the tree; 1,000 bootstrap values listed at supported nodes. NES, northern elephant seal (*Mirounga angustirostris*); CSL, California sea lion (*Zalophus californianus*); HS, harbor seal (*Phoca vitulina*); GFS, Guadalupe fur seal (*Arctocephalus townsendi*); SSL, Steller sea lion (*Eumetopias jubatus*); SO, sea otter (*Enhydra lutris*).

Phylogenetic analysis at the ITS1 locus established unequivocally that all 8 animals were infected with an identical sequence type that resolved as *N. caninum* (GenBank accession nos. PX529932–8) (Figure 2). For comparison, we included in the tree sequences recovered from other pinnipeds infected by *N. caninum*–like parasites that commonly circulate within a marine transmission cycle (*5*). We conducted PCR testing, determining 7 of the 8 *N. caninum*–infected animals had polyparasite infections with 1 or 2 other terrestrially sourced coccidian agents (*T. gondii*, *S. neurona*) in their tissues. Infected animals were 2 sea otters (*Enhydra lutris*), 2 harbor seals (*Phoca vitulina*), 1 northern elephant seal (*Mirounga angustirostris*), 1 Guadelupe fur seal (*Arctocephalus townsendi*), and 1 Steller sea lion (*Eumetopias jubatus*) (Appendix Table).

**Figure 3:**
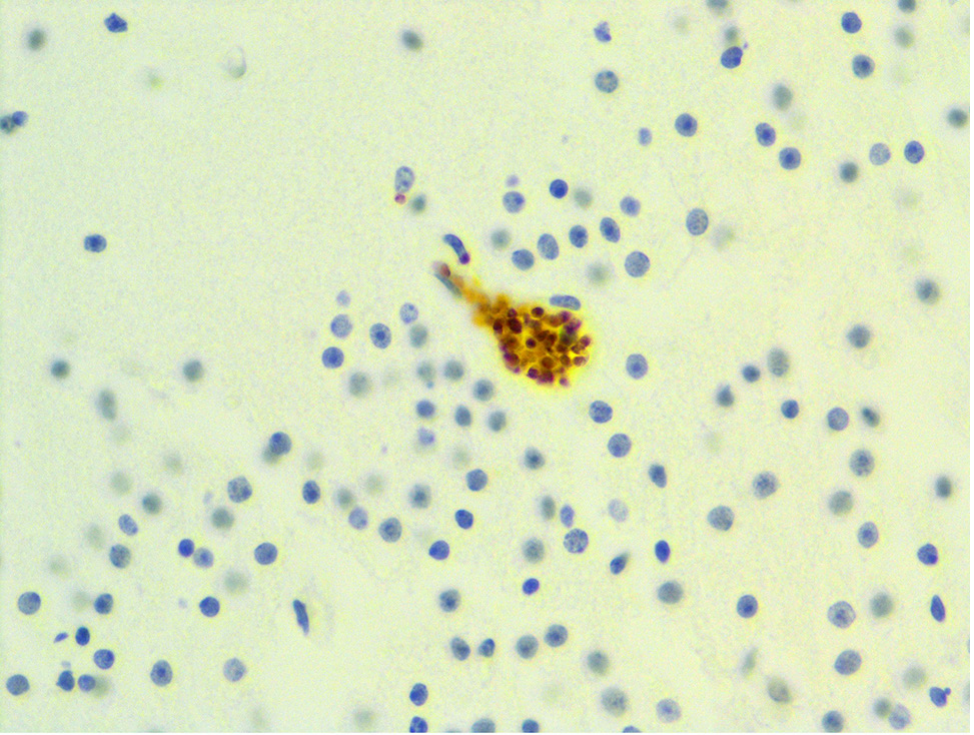
Immunohistochemistry of a harbor seal pup with *Neospora caninum* antigen localized within a neuron from study of *N. caninum* infection in marine mammals stranding in the northeastern Pacific Ocean region. Original magnification ×600.

We diagnosed nonsuppurative and necrotizing encephalitis (n = 2), meningoencephalomyelitis (n = 1), and meningoencephalitis (n = 1) by histology of the brain for 4 of 6 cases, including the pup and subadult harbor seals, adult Steller sea lion, and yearling California sea lion. We conducted immunohistochemistry in 4 encephalitic cases, observing *N. caninum* antigen in brain samples from the harbor seal pup and *T. gondii* and *S. neurona* (one or both) antigens in the other 3 animals (Figure 3). We found no pathognomonic lesions in this case series directly attributed to *N. caninum* infection. The cause of death for all 8 animals was independent of *N. caninum* infection (Appendix Table).

## Conclusion

We identified 8 animals from 6 marine mammal species unequivocally infected with *N. caninum*. Infection by this parasite alone was confirmed in 1 animal, a yearling California sea lion, and the infection represents another example of land-to-sea flow of a pollutagen of parasite origin. The contribution of this pathogen to marine mammal health is unknown. All infected animals we studied were from species with varying degrees of philopatry, with either short natal dispersal distances or extended oceanic to pelagic migrations. Seasonal haul out associated with pupping and breeding, coupled with accounts of wild canids scavenging at rookeries (*13*), provide insights into potential parasite introduction, persistence, and dissemination. For sea otters, bioaccumulation of parasites by invertebrate prey species may be inferred from observations of transmission dynamics with *T. gondii* (*4*). On the basis of gross and microscopic findings, we determined *N. caninum* infection to be unrelated to the proximate cause of death in the examined animals. Nevertheless, detection of *N. caninum* in a pregnant Steller sea lion, as well as in a harbor seal pup and northern elephant seal pup (weanling), is concerning. Recrudescence of latent infection during pregnancy contributes to parasite reactivation that results in vertical transmission in cattle and dogs (*6*), and we propose this phenomenon might be occurring sporadically in marine mammals.

Documented reports have confirmed land-to-sea transmission of protozoan parasites, and serosurveys for *N. caninum* have detected titers in marine mammal species. A recent review documents the global extent of land-to-sea pathogen flow (*14*). Seals and sea lions are monophyletic, members of the order Carnivora, and share a common ancestry with terrestrial canids. This evolutionary relationship may predispose these marine mammals to pollutagens defecated by canids. Cross reactivity with previously described nonpathogenic *N. caninum*–like parasites that circulate among pinnipeds in a marine transmission cycle, including coccidia types A and B (with California sea lions as definitive hosts) (*5*), may have contributed to false-positive results in prior serosurveys. The contribution of these *N. caninum–*like parasites to immune protection of hosts against *N. caninum* is unknown. In this limited series, we confirmed *N. caninum* infection in 2 sea otters, 2 harbor seals, 1 California sea lion, 1 northern elephant seal, 1 Steller sea lion, and 1 Guadelupe fur seal, none of which had been infected with previously described *N. caninum*–like parasites. Those findings extend the host range and ecologic niche for *N. caninum*. Infections were predominantly mixed with *T. gondii*, *S. neurona*, or both. Climate change, ecologic marine regime shifts, rural and urban development, weather events, and other environmental perturbations may lead to incursion of previously recognized land-based pathogens into the marine environment. 

In conclusion, our results indicate that further investigations to characterize the life history of *N. caninum* in the marine environment, the role of polyparasitism in disease manifestation, and the potential pathogenicity in susceptible host species are warranted. Whereas we cannot definitively attribute *N. caninum* as the cause of illness or death for the animals described here, future investigations of similar unexpected deaths, particularly those involving abortion storms among coastal breeding marine mammals in the northeastern Pacific, should consider molecular screening for this pathogen. 

Appendix Additional information for *Neospora caninum* in marine mammals stranding in northeastern Pacific Ocean region.
