# Supplementary material for: Neospora caninum Infection in Marine Mammals Stranding in Northeastern Pacific Ocean Region
Source: Emerg Infect Dis. 2026 Feb;32(2):277–80. doi: 10.3201/eid3202.251507 (PMC12928238; doi:10.3201/eid3202.251507)
Supplement: Appendix — Additional information for Neospora caninum in marine mammals stranding in northeastern Pacific Ocean region. [file 25-1507-Techapp-s1.pdf]

Article DOI: http://doi.org/10.3201/eid3202.251507

EID cannot ensure accessibility for supplementary materials supplied by authors. Readers who have difficulty accessing supplementary content should contact the authors for assistance.

Neospora caninum Infection in Marine Mammals Stranding in Northeastern Pacific Ocean Region

Appendix

Appendix Table. Summary of signalment, gross pathology, histopathology, molecular results, and assigned cause of death for a study of *Neospora caninum* in marine mammals stranding in northeastern Pacific Ocean region*

Case	Year	Species	Location	Age	Cause of Death	Neuropathology	Myocellular sarcocyst	Multisystemic inflammation	Nc	Sn	Tg
1	2007	GFS	WA	Yearling	Inflammation/ Infection	NA	NA	NA, autolysis	Ht	Br, Ht	Br
2	2007	HS	WA	Pup	Inflammation/ Infection	Marked meningoencephalomyelitis	NA	NA, hepatocellular hemosiderosis, lymphoid depletion	Br	Br, Ht	Br, Ht
3	2008	SSL	WA	Adult, pregnant	Inflammation/ Infection	Mild encephalitis, no antigen detected by IHC	NA	*Coxiella placentitis* with possible colonic rupture and sepsis	Br	none	Br, Ht, Skm
4	2008	SO	AK	Subadult	Inflammation/ Infection	Not examined	NA	Vegetative valvular endocarditis with sepsis	Br	none	Br, Ht
5	2009	HS	WA	Subadult	Inflammation/ Infection	Marked meningoencephalitis with florid intralesional protozoa	NA	NA, gunshot to the head with orchitis, bronchopneumonia and splenic and lymph node lymphoid hyperplasia	HO	Br, Tng, Skm	HO
6	2009	SO	BC	Adult	Hypovolemic shock	NA	Moderate	Myocarditis with intralesional bacteria and splenic rupture	HO	HO	none
7	2017	NES	CA	Pup	Emaciation, Inflammation/ Infection	Not examined	NA	Skin fold dermatitis and secondary sepsis	HO	none	HO
8	2017	CSL	CA	Yearling	Inflammation/ Infection	Mild gliosis and nonsuppurative meningitis	NA	Hindlimb subcutaneous abscessation with broncho and pleuropneumonia	HO	none	none

*NA, tissues examined with no apparent lesions; Ht, heart; Br, brain; Skm, skeletal muscle; HO, tissue homogenates. GFS, Guadalupe fur seal; HS, harbor seal; SSL, Steller sea lion; SO, sea otter; NES, Northern elephant seal; and CSL, California sea lion. Molecular results: Nc, *Neospora caninum*; Sn, *Sarcocystis neurona*; Tg, *Toxoplasma gondii*; WA, Washington; AK, Alaska; BC, British Columbia; CA, California.
